# A Hybrid 3D-2D Image Registration Framework for Pedicle Screw Trajectory Registration between Intraoperative X-ray Image and Preoperative CT Image

**DOI:** 10.3390/jimaging8070185

**Published:** 2022-07-06

**Authors:** Roshan Ramakrishna Naik, Anitha Hoblidar, Shyamasunder N. Bhat, Nishanth Ampar, Raghuraj Kundangar

**Affiliations:** 1 Manipal Institute of Technology, Manipal Academy of Higher Education Manipal, Manipal 576104, India; roshannaik10091983@gmail.com; 2 Kasturba Medical College, Manipal Academy of Higher Education Manipal, Manipal 576104, India; ortho.dr.nishanth@gmail.com (N.A.); raghuraj.sk@manipal.edu (R.K.)

**Keywords:** 3D-2D registration, digitally reconstructed radiographs, iterative control point registration, optimization, pedicle screw insertion, similarity metric

## Abstract

Pedicle screw insertion is considered a complex surgery among Orthopaedics surgeons. Exclusively to prevent postoperative complications associated with pedicle screw insertion, various types of image intensity registration-based navigation systems have been developed. These systems are computation-intensive, have a small capture range and have local maxima issues. On the other hand, deep learning-based techniques lack registration generalizability and have data dependency. To overcome these limitations, a patient-specific hybrid 3D-2D registration principled framework was designed to map a pedicle screw trajectory between intraoperative X-ray image and preoperative CT image. An anatomical landmark-based 3D-2D Iterative Control Point (ICP) registration was performed to register a pedicular marker pose between the X-ray images and axial preoperative CT images. The registration framework was clinically validated by generating projection images possessing an optimal match with intraoperative X-ray images at the corresponding control point registration. The effectiveness of the registered trajectory was evaluated in terms of displacement and directional errors after reprojecting its position on 2D radiographic planes. The mean Euclidean distances for the Head and Tail end of the reprojected trajectory from the actual trajectory in the AP and lateral planes were shown to be 0.6–0.8 mm and 0.5–1.6 mm, respectively. Similarly, the corresponding mean directional errors were found to be $4.9^{0}$ and $2^{0}$. The mean trajectory length difference between the actual and registered trajectory was shown to be 2.67 mm. The approximate time required in the intraoperative environment to axially map the marker position for a single vertebra was found to be 3 min. Utilizing the markerless registration techniques, the designed framework functions like a screw navigation tool, and assures the quality of surgery being performed by limiting the need of postoperative CT.

## 1. Introduction

Currently, Pedicle Screw Insertion (PSI) is a common type of surgery performed to fix fractures, decompression or deformities of vertebrae. In PSI surgery, once the entry point (facet joint and transverse process junction) for screw insertion is identified, using a high-speed burr, posterior cortical breaching is performed. This is followed by insertion of gearshift probe to a depth of 20 mm to 25 mm, pointing the tip in a lateral to medial direction, and then insertion is continued to the desired depth [1]. During this pilot hole creation, a feeler probe or ball-tipped probe is used to feel the entrance into the cancellous bone of the pedicle and other bony regions of the vertebra. If any breaching is palpated, then the trajectory is redirected with a suitable degree of instinctive correction. For easier screw insertion, pilot hole is redefined through a process known as undertapping. Threading of the screw is performed using a multiaxial screwdriver. A series of radiographs is acquired during the pilot hole creation in the AP and/or lateral direction to confirm the position of the surgical probe relative to the target region.

PSI is associated with a wide variety of postoperative complications due to vertebra’s complex structure and its proximity to neighboring tissue regions. In the PSI procedure, varieties of postoperative complications exist—these include injuries to the nerve root, spinal cord or vascular systems such as azygos vein, intercostal artery, inferior vena cava in the thoracic region, and mainly the aorta and common iliac vessel in the lumbar spinal region [1]. In addition, cerebrospinal fluid leak, visceral injury, and pedicle fracture are some of the other types of listed postoperative complications [2]. The pedicle screw navigational error may lead to the pedicle’s medial or lateral breaches and sometimes anterolateral perforations [3]. Furthermore, a breach of the pedicle can damage nerve roots, the dural sac, vascular structures, and pleura [2]. The level of breaches can be measured in the axial and sagittal planes of vertebral anatomy. A screw navigational error grading system is referred for qualitative and quantitative evaluation of these breaches. The grading scale includes grade A to grade E breach depth ratings classified in the step interval of 2 mm—measuring the degree of deviation of the screw from the ideal screw trajectory [4,5].

A better screw trajectory plan relative to patient-specific vertebral morphology leads to a gratifying PSI surgery. The screw insertion plan includes assessment of vertebral morphology and pedicular dimension analysis such as its diameter, length and angle. Additionally, entry point, pedicular center point, and the target region inside the vertebral body are identified. The ideal screw trajectory is planned along the cylindrical axis of the pedicle [6]. However, alternative minimal invasive procedures are being practiced in PSI wherein the trajectory entry point and angle of insertion are planned according to the target vertebral level. Furthermore, the anchorage of the pedicle screw depends on the bone mineral density, screw design and dimension, and pilot hole preparation method followed [7,8,9]. The postoperative CT images can be referred to measure the deviation of the actual trajectory in comparison to the planned trajectory.

According to the study conducted for quality practice, freehand-based PSI surgeries strongly recommend experienced surgeons over novice ones [3]. In addition, due to the unavailability of the 3D fluoroscopy imaging system, higher imaging cost, dosage reduction, reproducibility and spatial occupancy issues over the years, different Computer Assisted Surgeries (CAS) have been designed specifically to assist Pedicle Screw Surgeries (PSS) [10,11,12,13,14,15]. These CAS systems have improved the quality and success rate in PSS. However, there are variety of limitations in existing designed tools. A conventional navigation tool has a variety of issues such as the requirement of reregistration due to the marker movements, imperceptible Field Of View (FOV), the necessity of direct line of sight in the presence of optical-based navigational trackers, cost ineffectiveness of CAS systems, interference and lack of robustness issues in magnetic-based tracking systems. These limitations hold back their application in the intraoperative environment [16,17,18].

The previously described conventional PSI procedure is completely based on manual feedback. In this procedure, the target region cannot be directly visualized and fiducial marker or tracker-based navigation tools are also not widely practiced. Hence, the quality of the surgery that surgeons experience is truly instinctive. In addition, because of the limited vision provided by the intraoperative radiographs, surgeons could perceive the positioning of the screws incorrectly. Furthermore, the practice has too much exposure to the X-ray radiations due to the necessity of acquiring a series of images intending to confirm the navigation of the surgical tool all through the posterior entry point to the anterior most targeted point. Hence, to estimate 3D screw pose in the intraoperative environment utilizing minimal intraoperative X-ray images, a 3D-2D image registration-principled semi-automated navigation tool is designed. The proposed framework addresses the challenges encountered during PSI surgery by naturally fitting in line with the existing PSI procedures usually practiced in many local hospitals.

**Figure 1:**
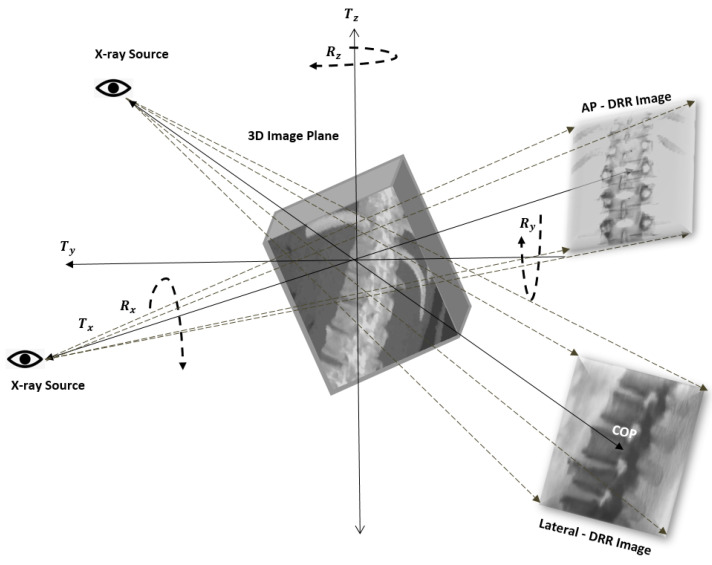
Depiction of projection image generation in the Anterior–Posterior (AP) and lateral directions during the intensity-based 3D-2D registration.

The image registration-based navigation systems had been designed to function as a navigation tool in the operation theater [19,20]. The 3D-2D intensity-based image registration system is one of the popular image registration technique applied in medical image registration alternatives to feature-based and gradient-based image registrations [21]. The generation of projection images during the intensity-based 3D-2D registration is depicted in the Figure 1. For pedicle screw trajectory registration, we propose a novel hybrid registration framework that incorporates a combination of Iterative Control Point (ICP) and Digitally Reconstructed Radiographs (DRR)-based registration techniques. The contribution of our work includes designing of a variant of ICP based 3D-2D landmark registration, DRR image generation and evaluation of registration framework. The ICP-based registration is preferred over intensity-based registration due to its faster pose estimation and large capture-range benefits. The framework utilizes the DRR generation technique in the various stages of registration to verify and visually validate the accuracy of control point registrations. After the mapping of the trajectory from intraoperative X-ray image to preoperative CT(pCT), its pose is reprojected back to the intraoperative X-ray image coordinates for the evaluation [22]. The framework performs trajectory mapping to a clinical acceptance precision conforming to the intraoperative target registration standards by limiting the necessity of postoperative CT.

## 2. Literature Survey

The literature survey includes intensity-based 3D-2D registration principled PSI navigation tools designed for the clinical applications and their pros and cons. In addition, an alternative to intensity-based 3D-2D registration technique, i.e., Iterative Control Point 3D-2D registration—a sub-type of feature-based registration and its significance and limitations in a clinical application perspective are discussed. In addition, we have discussed the latest navigation technologies that would be ubiquitously utilized in Orthopaedics surgeries in the coming future.

### 2.1. 3D-2D Registration Principled Surgical Tool Navigation Systems

The 3D component model of the pedicle screw was designed, and its position from multiple radiographs was registered onto 3D CT to function as a navigation tool in PSS [23]. Three intraoperative radiographs were acquired to register anatomy and Pedicle Screw (PS) details. The geometrical registration accuracy of screws modeled after parametric models and exact component-specific CAD models was compared. The framework was designated to provide quality assurance of the surgical product by providing visualization of screw position relative to trajectory planning and pedicle acceptance window. The conducted study was supportive for faster intraoperative applications but lacked study on the patient dataset.

Uneri and team designed a tool to overlay scanned and parameterized model of probe tool and pedicle screw onto pCT through known component-based registration [24]. In this surgical guidance, target registration accuracy was investigated as a function of projection view angle. The author claimed that between the reference images angle separation of $10^{0}$ was sufficient enough to attain Target Registration Error (TRE) of 2 mm. As compared to electromagnetic tracker-based localization, the designed tool provided better TRE—achieved with 95% confidence for angular separation of at least $10^{0}$ [25]. In another work by Uneri et al. [26], a near real-time PS navigation guidance system was developed, and the accuracy of screw navigation after incorporation of various methodology was tested. After patient pose estimation from multiple (AP, Oblique and Lateral) radiographs, 3D screw pose was estimated within the patient reference frame. The screw model details such as length and diameter of shaft, tip and cap information were mapped on the CT dataset. The registration accuracy was investigated under the calibrated and uncalibrated conditions of the imaging system. In addition, registrations of multiple pedicle screws under the sequential and simultaneous registration conditions were explored. Both in phantom and patient datasets, the simultaneous-calibrated condition resulted in the best TRE. Joint pose estimation of patient and screw increased the computation time because of the increased convergence time that was required for multiple parameters to settle. Besides, registration accuracy did not improve significantly.

To simulate the spinal fixation procedure, a two-stage deformable registration framework was designed to register device models of K-wire and spinal fixation rods on CT image [27]. The X-ray-CT registration framework utilized three radiographs for anatomy registration, and then deformable registration of K-wire polygonal meshes was performed. The framework functioned like a 3D navigation system without utilizing any tacker or fiducial marker. The study was conducted on the phantom model additionally by considering exceptional situations of medial and lateral breaching cases.

An intraoperative image guidance system based on 3D-2D registration was designed to provide visualization of pedicle screw navigation in the CT image [15]. The Registration accuracy was measured under both distorted and undistorted fluoroscopic imaging conditions for screws made of different materials and lengths inserted over different vertebral levels. Various clinical scenarios such as medial and lateral breaches were simulated in the study model, and the presence or absence of these breaching situations were subjected to test the robustness of the designed system. The designed system provided better identification and visuals of these scenarios in comparison to the surgeon’s interpretations. The work limits its experiment on the nonclinical dataset in which the influence of soft tissue and skeletons was neglected.

A Convolutional Neural Network (CNN)-based deep learning framework was designed for automatic PS segmentation and pose estimation [28]. Initially, the screw region was segmented from an X-ray image; then, using biplanar X-ray imaging knowledge the pose of the screw in 3D space was estimated. The FCN (Fully Convolutional Network) architecture was used to segment the screws from the X-ray images. Later, its center axis was finally mapped to CT by corroborating C-arm calibration parameters. The framework was trained by creating a database of projection images derived after utilizing different PS models at variable 3D poses. The registered trajectories were evaluated relative to the reference axis in the CT reference frame. In another work by Esfandiari et al. [29], various combinations of similarity and optimization schemes were applied in the context of 3D-2D intensity-based PS registration. The Gradient Correlation–Powell optimizer combination pair provided the widest capture range and was found to be least sensitive to the X-ray image contrast. The implant projections have reduced the capture range. The study was performed on the simulated data by simulating variable clinical scenarios and lacks experiment with the real intraoperative images.

On the other hand, to measure 3D Acetabular cup orientation after the Total Hip Replacement (THR) surgery, a 3D-2D registration-based tool was designed. The tool utilizes a single 2D postoperative AP radiograph to get registered with 3D preoperative CT image [30]. The registration error between the registered implant and its actual postoperative CT pose was computed. The experiment was conducted on a plastic pelvis phantom. It was claimed that registration accuracy could have been improved by referring to multiple radiographs. In addition, postoperative measurements were inaccurate due to the scattering effects of the head region of the implant.

In another work, to assist pelvic screw placement and patient-specific screw trajectory planning, and to provide surgical guidance and quality assurance—a trackerless registration of deformable K-wire device was presented [31]. The automatic trajectory planning was traced after the creation of the Active Shape Model of patient-specific pelvis. The registered trajectory was overlaid on CT to function as a navigation tool, and its pose precision was evaluated against the planned trajectory. The deformable K-wire registration provided better registration accuracy than rigid optical based tracking.

### 2.2. ICP Based 3D-2D Registration in the Clinical Applications

As an alternative to intensity-based 3D-2D registration, a point set-based 3D-2D registration—a sub type of feature-based registration was proposed. This point set registration includes an Iterative Control Point (ICP) registration type wherein the target points are registered with source point sets. These registration techniques are popular for their simplicity and low computational complexities [32]. The ICP principled registration techniques were preferred majorly in 3D-2D registration of vessels in coronary artery surgery or neurointerventions [33,34]. ICP based 3D-2D registration and its variants have been explored in both rigid and nonrigid clinical registration problems.

To support in neurointerventions a framework was designed by McLaughlin et al. [35] wherein the performance of ICP-based registration was compared with intensity-based registration. The ICP based registration approaches were found to be suitable over gradient-based registration when 3D-2D point pairs were not erroneous. The speed of computation and capture range was found to be superior to its counterpart. In another variant of 3D-2D point-set registration, vessels from cerebral angiogram were registered [36]. To speed up the registration, a global optimal rotational search was performed in individual blocks of transnational space. The framework outperformed state-of-the-art’s local and global search methods in terms of speed of computation and rotational error measurements. For cerebral vessel registration, a two-step double plane registration was performed by Fu et al. [34], and the performance of the method was compared with the state-of-the-art point set registrations. A novel objective function was defined wherein the lines joining the target 2D point and source projection center formed a consensus pair with corresponding 3D point set on optimal registration. The double plane registration has improved the rotational and transnational error measurement over single plane registration as performed by Liu et al. [36].

To overcome the issues of deep learning-based registrations such as the data dependency and poor generalization ability, and to avoid high computational complexity associated with intensity-based 3D-2D registration, a hybrid registration framework possessing ICP-DRR-based registration modules is proposed. The proposed framework incorporates landmark-based registration to naturally fit in line with the existing surgical practice wherein surgeons intuitively map target points between the interimaging datasets. The framework bypasses the requirement of consecutive DRR image generation during the optimization stage of conventional intensity-based 3D-2D registration, hence making the system applicable in faster intraoperative applications without necessitating any GPU (Graphical Processing Unit) or parallel computation platforms.

### 2.3. Navigation Systems Based on Augmented Reality and Robotic Assistance

Without acquiring intraoperative radiographic images HoloLens—a Head Mounted Display (HMD) based visual augmented reality-based navigation systems were developed to improve the accuracy and reproducibility in orthopaedic surgeries [37,38,39]. These systems address the issues encountered by conventional optical-based registration methods by providing in situ visualization by integrating markerless tracking and registration with existing Augmented Reality HMDs (AR-HMDs). These systems can be classified as video see through or optical see through systems based on the surgery environment being supplied to the display device. The functioning of HoloLens in surgical practice is based on the following steps. The HoloLens employs simultaneous localization and mapping algorithms to locate the sensors within its mapped environment. The two front cameras of HoloLens locate the 3D position of markers using the triangulation technique. Before surgical incisions, the pose correspondence is established between markers affixed to the surgical tool and markers placed on the patient [40]. This HoloLens possesses depth sensors that can be utilized during patient pose estimation. The major advantage of this system is that it abolishes monitor-based visualization without compelling eyesight shifts from the target region. The system provides preoperative planning on intraoperative anatomy to support live corrections during the incisions. The studies in this direction are in the initial stage and are majorly limited to phantom, CAD and other 3D anatomical models rather than clinical applications.

Alternatively, to practice pedicle screw insertion, various robot-assisted surgery systems have been developed [39,41]. Most of these systems utilize optical cameras to track optical markers affixed to surgical tools relative to the marker fixed to the patient anatomy. Some of these systems are facilitated to get integrated with existing 3D intraoperative imaging devices or 2D intraoperative fluoroscopic devices to support in situ visualization. The intraoperative trajectory localization of these robot-assisted surgery systems is dominating over other fluoroscopic-based navigation systems and has minimized breaching rates measured as per the Gertzbein–Robbin scale. Such systems are well preferred under zero intraoperative radiation exposure and minimal invasive surgeries when the required difference in surgical procedural efficiency between novice and experienced surgeons is suboptimal.

## 3. Materials and Methods

**Figure 2:**
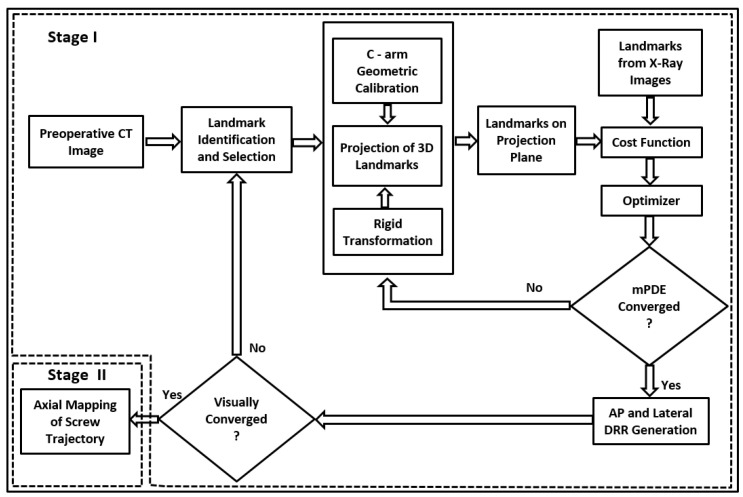
Workflow of hybrid ICP-DRR based 3D-2D registration framework for pedicle screw registration.

The registration framework is divided into two stages, i.e., anatomical landmark registration followed by trajectory registration. The stage I, as depicted in Figure 2, is a framework for ICP-DRR-based registration designed for intraoperative vertebral pose estimation. The anatomical landmark registration estimates the transformation parameter of the intraoperative imaging device at which the respective vertebra is projected. For AP and lateral X-ray image space to C-arm—pCT pose estimation, a variant of ICP based 3D-2D registration is followed. Due to postural differences between the preoperative and intraoperative environments, individual vertebrae undergo variable degrees of deformation. Hence, from the pCT dataset, the target vertebra is cropped, and individually registered. This type of single-level registration is locally rigid but globally deformable, localizing the vertebral centroids with better accuracy, especially when the patient’s pose differs according to the image acquisition protocol [42]. Besides, during the pilot hole creation, it is assumed that there would be trivial structural deformation within the rigid vertebra. Subsequently, in stage II, the marker landmark positions are back-projected to axial pCT for trajectory mapping. Ultimately, on convergence, the registration loop is terminated, and the accuracy of the trajectory path is evaluated on a 2D plane. To clinically validate the precision of registration along with mean Projection Distance Error (mPDE) measurements, DRR images possessing optimal match with intraoperative X-ray images are generated.

### 3.1. Stage I—Anatomy Landmark Registration in AP and Lateral Planes

The stage I process includes preprocessing of pCT, trajectory planning in pCT, C-arm camera modeling, DRR generation, and cost function optimization utilizing certain anatomical landmarks from 3D and 2D planes.

#### 3.1.1. Preprocessing and Trajectory Planning

The pCT and X-ray images are acquired at resolution as mentioned in the Appendix A Table A1. Prior to the registration initiation, target vertebral region subjected to PSI was cropped from both pCT and X-ray images. For screw size selection and screw trajectory planning specific to the target vertebra, the following procedure is practiced. A midline from the posterior end to the anterior end is drawn on the mid axial slice of the target vertebra such that the line axially bisects the vertebra symmetrically. For a screw trajectory plan, a line passing through the screw entry point, the pedicle cross point and the trajectory endpoint known as Transverse Pedicular Angle (TPA) is defined. The perpendicular intersection of TPA and pedicle transverse width is considered as a pedicle cross point. The pedicle cross point is always identified as the center point of the pedicle in a slice having maximum pedicular width when viewed in all the planes. The trajectory is planned such that it could provision initiation in the lateral direction; then, on advancing the probe, the axis would get penetrated medially to the desired depth passing through the pedicle cross point. The insertion angle depends on the pedicle angle which varies according to the vertebral level, screw purchase level, and insertion point selection [1,43,44]. To avoid wrong level surgery, prior to PSI, surgeons performed vertebrae-level identification in both X-ray and pCT images. Once the target vertebra is exposed at the posterior end, the entry point is identified such that the burr placed at the pedicle periphery would attain 2-o’ or 3-o’clock and 10-o’ or 9-o’clock position with the right and left pedicle-lamina intersection point of respective vertebra [1]. The screw axis is maintained parallel to the body endplates and resides approximately below one-third of the vertebral body height from the superior body endplate.

#### 3.1.2. C-Arm Camera Model and Mathematical Preliminaries to Project a 3D Point onto a 2D Plane

To generate the DRR images of pCT in various directions, the movement of the C-arm camera is modeled utilizing the extrinsic and intrinsic matrix of the C-arm imaging device. The extrinsic parameter includes translational ($T_{x},T_{y},T_{z}$) and rotational parameters ($\theta_{x},\theta_{y},\theta_{z}$). The 3D-2D point set mapping is performed using a pinhole camera model designed from the calibrated C-arm imaging device specifications. This includes intrinsic parameters such as focal length, Center Of Projection (COP), and pixel size in the horizontal and vertical direction of the detector plane.

**Figure 3:**
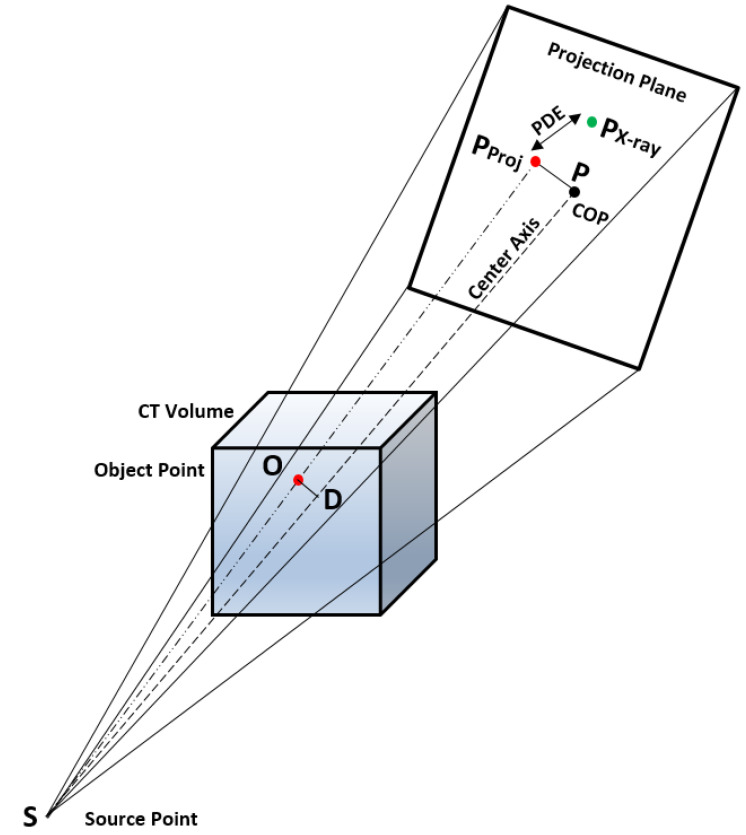
Depiction of C-arm camera model to project 3D point on 2D projection plane and PDE measurement.

The landmarks from the 3D plane to the 2D plane can be projected as follows. Consider a source point $\vec{S}$, object point $\vec{O}$, projected point $\vec{P_{Proj}}$ and Center Of Projection (COP) point $\vec{P}$, as depicted in Figure 3. The vectors utilized to project a 3D pCT point on a 2D detector plane are defined as below.

Sourceto object vector

(1) $$\vec{SO}=\vec{O}-\vec{S}$$

Source to center point of projection vector

(2) $$\vec{SDD}=\vec{SP}=\vec{P}-\vec{S}$$

If *D* is the projection point of object vector on the center axis $\vec{SP}$, then intersection point $\vec{D}$ on $\vec{SP}$ can be found at “*t*” as defined in Equation (3).

(3) $$t=\frac{\vec{SP}⊙\vec{SO}}{\left|\right|\vec{SP}{\left|\right|}^{2}}$$

Projection of object point “$\vec{O}$” on the center axis

(4) $$\vec{D}=\vec{S}+\left(\vec{P}-\vec{S}\right)*||t||$$

Source to object projection point distance vector on center axis

(5) $$\vec{SD}=\vec{SOB}=\vec{D}-\vec{S}$$

Magnification factor

(6) $$M=\frac{\left|\right|\vec{SDD}\left|\right|}{\left|\right|\vec{SOB}\left|\right|}$$

Projection coordinates on the detector plane

(7) $$\vec{P_{Proj}}\left(x,y,z\right)=\vec{P}\left(x,y,z\right)-||M||*\left(\vec{D}-\vec{O}\right)$$

The algorithm can be summarized in three simple steps. Initially, object point to center axis distance is measured ($\vec{OD}$), later magnification factor is measured (*M*) and finally the magnified length between the projected point and the COP in the projection plane is computed.

#### 3.1.3. Generation of DRR Image

The projection in the AP and lateral directions is derived from pCT using the raycasting technique, and these projection images are referred to visually verify the estimated registration parameters before trajectory mapping. The landmark points are overlaid on the DRR images to visually validate the estimated transformations relative to the real radiograph images. In the raycasting process, Linear Attenuation Coefficients (LAC) of anatomical tissues are cumulated along the virtual X-ray in the AP and lateral directions, as depicted in Figure 1. The intensity of a detected pixel is computed after integrating LAC along the ray as per the Equation (8) [45].

(8) $$I=I_{0}exp^{-\sum_{i=1}^{n}\mu_{i}\left(E_{C}T\right)*l_{i}}$$

where *I* is the projected pixel intensity through the tissue volume, $I_{0}$ is the X-ray intensity of X-ray photon directly received by the detector without undergoing any degree of attenuation, $\mu (E_{C}T)$ is the LAC of tissue voxel—computed relative to water attenuation ($\mu_{water}\left(E_{C}T\right))$ at CT energy ($E_{C}T$) from Hounsfield Unit(HU) as defined in Equation (9), *l* is the voxel intersection length and *i* denotes voxel index [45].

(9) $$\mu \left(E_{C}T\right)=\frac{1000+HU_{x}}{1000}.\mu_{water}\left(E_{C}T\right)$$

#### 3.1.4. Anatomical Landmark Identifications and Registration

The datasets of two patients subjected to multilevel PSI are collected utilizing Philips BV Endura and Ziehm 8000 C-arm imaging devices. The dataset specifications are given in the Appendix A. The registration framework is executed after anatomical landmark identification and selection of an initial point of registration. The intraoperative radiographs are acquired such that the vertebral body endplates appear as a sharp line with symmetric pedicles rostral to the endplates and the spinous process is in the midline of vertebra [1]. However, in some cases, vertebral levels miss an End On View (EOV) resulting in poor landmark registration and trajectory angle mapping (Vertebra L5 in our study). The initial point of registration is selected such that the majority of the anatomical regions in the initially generated DRR image appear as in the X-ray image. To estimate the vertebral pose in the AP and lateral planes, based on surgeons’ 3D-2D landmark correspondence knowledge certain landmarks are identified in X-ray and pCT images. To illustrate, in the AP view of T11 and L1, the centroids of pedicles, inferior end of the spinous processes and vertebral body centroids are preferred. In the lateral direction, four vertebral body corners and a vertebral centroid are chosen as target landmarks to be registered. The landmark selection varies depending on whether the vertebral body image in AP or lateral view has a single bounded edge visible after EOV or multiple edges. If in the AP/lateral X-ray view, when both anterior and posterior vertebral body endplate edges are visible, the center points of the curved edges are selected as control points to be registered (L3). Such changes in the landmark selection guides in optimally registering out-of-plane rotational parameters of the vertebra. Due to the ill shape of the fractured vertebra ‘L4’ and unclear endplate edges of vertebra ‘L5’, both landmark identification and registration were challenging, and hence resulted in moderate registration accuracies as described in the Result and Discussion sections. On optimal registration, the transformation parameter of the C-arm pose projects the target vertebra at which it was acquired in the intraoperative environment relative to the preoperative CT coordinate system.

#### 3.1.5. Cost Function

The Euclidean distance between projected and radiograph landmarks is considered as cost function to be minimized during the optimization process. The mean value of PDE (mPDE) over “*N*” different landmarks is computed as defined in Equation (10). On optimal registration, the transformation “$T_{Reg}$” transforms a set of “*N*” landmarks from 3D ($P_{CT}$) to AP or lateral planes, as defined in the Equation (11).

(10) $$mPDE=\frac{1}{N}\sum_{k=1}^{k=N}T_{Reg}.P_{CT_{k}}-P_{Xray_{k}}$$

(11) $$P_{Proj}\left(x,y,z\right)=T_{Reg}.P_{CT}\left(x,y,z\right)$$

#### 3.1.6. Optimization

The designed framework employs Covariance Matrix Adaptation Evolution Strategy (CMAES)—an iterative nonlinear derivative-free optimization algorithm. The algorithm is designated to estimate transformation to attain the best mapping between projected and real X-ray image landmarks. In this algorithm, search space parameters are normally distributed and are represented using a covariance matrix. The mean and variance parameters determine the shape and size of the covariance matrix of distributed samples.

In each iteration, from parent sample points, new search points called offspring points are derived and selected based on fitness measures through a process known as recombination and selection. The offspring points from the current generation would be assigned as the parent sample points for the next generation if its fitness value is maximum among other offspring sample points. The CMAES is a rank-based selection wherein in every single generation, “μ” samples are selected from “λ” sample population by iteratively adjusting strategy parameters, covariance matrix, and step size [46]. The transformation parameters are chosen as objective vectors, whose values are tuned iteratively across generations minimizing the mean Euclidean distance metric. The Euclidean distance is computed between a set of projected and target landmark points as described in Equation (10).

The CMA is performed to enhance the probability of reproducing the successful search direction successively across generations. The CMA employed in our work incorporates rank one and rank—μ update strategy. The rank one update estimates the long-term average change that has to be maintained across successive generations. The rank μ update increases the variance of search direction based on the maximum likelihood estimation of successful localization [47].

In addition to covariance matrix adaptation, step size control is utilized to travel the rugged search space in a direction of the search space of interest. The step size control is performed by utilizing the evolution path. The evolution path in Evolution Strategy (ES) based optimization considers sample mean and variance from the current and previous generations. When samples get mutated across generations, the evolution path reveals correlation information. This concept of the evolution path updates the covariance matrix in a significant way by exploiting the correlation between the consecutive steps. Suppose if successively selected mutations are parallel correlated, the evolution path will be longer, signifying that search direction is the same, and consequently step size is increased for faster convergence. Otherwise, when they are anti-correlated, short step sizes are considered. This process is known as cumulative step size adaptation [46,48].

The mean and variance of transformation parameters are selected from the knowledge of the difference between the initial projected image and the real X-ray image. The larger variation in sample distribution may converge the objective function to local maxima driving the localization to inappropriate transformation. Hence, mean and variance are chosen such that the generated DRR image would include major anatomy as appearing in the real X-ray image. The total number of evaluations and population size are set to 2000 and 50, respectively. The rest of the parameters are defined as per the work Hansen et al. [46]. In the optimization stage of stage-I, the convergence criteria are set to zero mPDE or to a total number of evaluations.

### 3.2. Stage II—Trajectory Registration

The stage II includes trajectory axial mapping on pCT at the registered vertebral pose. After the pilot hole creation, a radio-opaque pedicular marker is placed inside the pilot hole, and on optimal landmark registration, the pose of this marker is axially mapped on pCT in the world coordinate system utilizing the designed C-arm camera model. The head and tail ends of the radio-opaque pedicular marker of length 3 cm are back-projected onto pCT, and intersection points of virtual X-rays are computed to identify the entry point and target point. The accuracy of trajectory mapping depends on the accuracy of anatomical landmark registration. On imprecise pose estimation, the positions of the landmarks can be varied about their previous markings or different landmark combination sets can be selected. Because of registration errors, mainly due to imprecise landmark identification and localization, and imprecise anatomical landmark mapping between the AP and lateral X-ray images, the trajectories in AP and lateral directions did not get intersected. In such a scenario, the midpoint of the shortest distance between the trajectories is identified as the corresponding localization point. The trajectory evaluation is performed on the intraoperative radiographic planes, and the registration accuracy is evaluated based on three surgeons’ feedback.

## 4. Evaluation

To evaluate the precision of 3D-2D registration, various regularizations were followed. These standardizations define the evaluation protocol, evaluation criteria and definition of ground truth registration, etc. [21]. The margin of error for pedicle screw placement was geometrically represented in the 3D plane by Rampersaud et al. [49] through modeling the pedicle and screw diameters in the cylindrical form. According to this analysis for image-guided pedicle screw placement, the maximum permissible translational and rotational error in 3D can be as high as 1 mm and $5^{0}$, respectively. In the 3D plane, the navigation system designated for clinical PSS can result in a target localization error of 2 mm [23]. In some of the research by Goerres et al. [31] and Uneri et al. [24], the registered trajectory accuracy was measured in a 3D plane relative to the planned trajectory. In the absence of postoperative CT data and on an unavailable fiducial marker or tracker-based ground truth registration data, we evaluated the precision of registration in the 2D plane [22]. The stage I results are clinically validated by generating projection images at the registered anatomical landmarks possessing optimal resemblance with that of real intraoperative images [29].

After the trajectory mapping in the 3D plane, the registered trajectory is back-projected on the 2D plane to measure its pose relative to the marker pose. In our framework evaluation, seven different metrics are considered. This includes errors in localizing the trajectory entry and target positions, trajectory angles in two planes, and the difference in trajectory lengths. The Euclidean distance and geometrical angle error measurements are performed separately in the AP and lateral planes. To strengthen the previously defined assessments and for reliability, all the metrics are evaluated by the surgeons. Furthermore, after the stag-I registration, the screw positions from AP and lateral X-ray images are overlaid on corresponding DRR images. This information strengthens surgeons’ interpretation of screw position in 3D plane relative to the vertebral anatomy as displayed in 2D image. This test supports detecting the possibilities of any breaches that can be sensed utilizing AP and/or lateral C-arm images in the intraoperative environment without postoperative CT images [50].

## 5. Results

**Figure 4:**
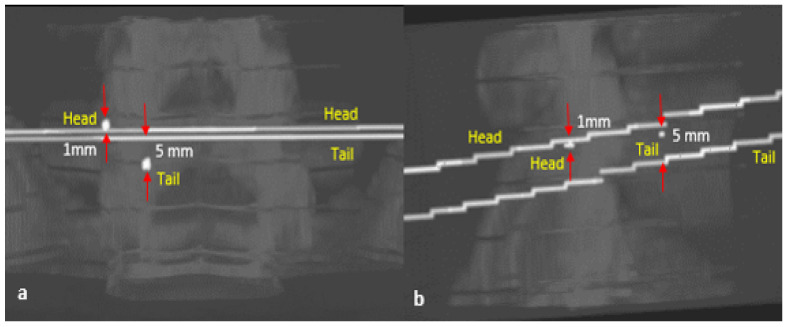
The screw trajectory backtracing from AP (**a**) and lateral (**b**) planes to pCT in L5 vertebra. The gap between the trajectories in different planes is measured and displayed—Source: Orthopaedics and Radiology Department—KMC Manipal.

**Figure 5:**
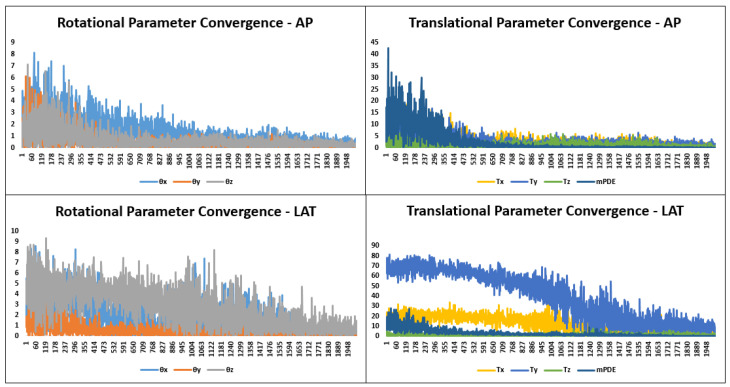
Transformation parameter and mPDE convergence during the optimization in AP and lateral planes are displayed about the final estimation over successive number of evaluation.

**Figure 6:**
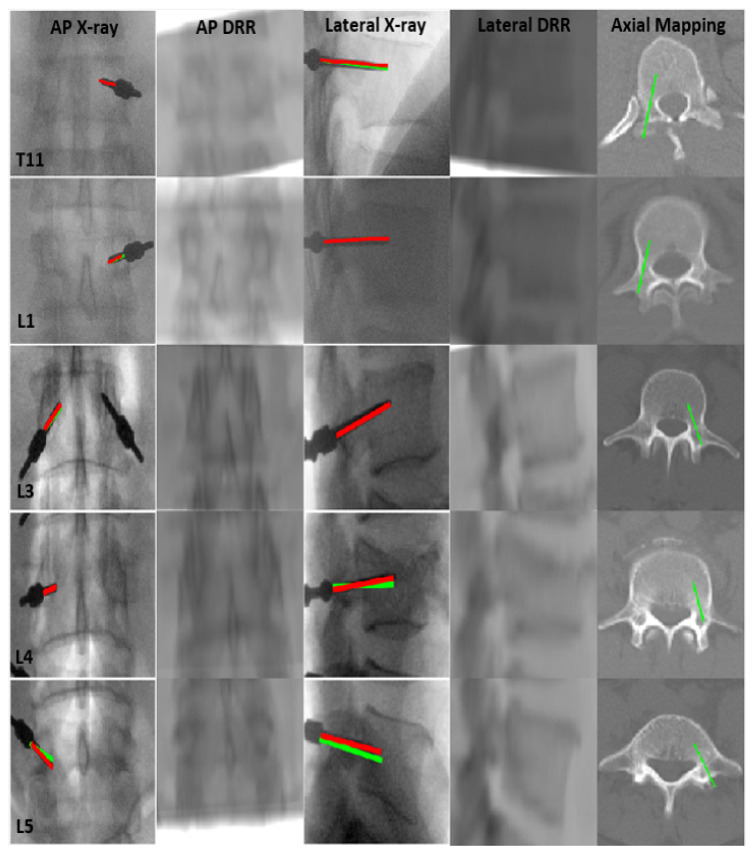
Clinical validation of pedicle screw trajectory registration: Red and Green lines in Columns 1 and 3 are the center axis of true and reprojected trajectories displayed on AP and lateral radiographs, respectively. Columns 2 and 4 display DRR images generated at the optimal anatomical landmark registration point in the AP and lateral planes, respectively. Column 5 displays axial trajectory mapping on pCT at optimum registration point of different vertebral levels—Source: Orthopaedics and Radiology Department—KMC Manipal.

On optimum registration, the mPDE measurement stagnated at optimum value and registered the landmarks with better accuracy. After the anatomical landmark registration, the DRR image projected the same anatomical region as that of a real X-ray image possessing an optimal match in terms of structure, pose and shape. On backprojection, the marker’s head and tail ends of trajectories did not get intersected due to imprecise one-to-one landmark alignment in the AP and lateral directions. The distance between the unconnected trajectories for different vertebrae is shown in Table 1 and pictorially displayed in Figure 4 for one of the vertebra. In the case of the L5 vertebra, the gap between the trajectories was greater, which in turn impacted on poor trajectory angle registration, as reflected in Table 2. The Figure 5 displays the convergence of rotational and translational parameters along with mPDE measurements about the final estimation. The Figure 6 displays the screw trajectory’s axial mapping in pCT image and DRR images in the AP and lateral planes generated to clinically validate estimated vertebral pose. Since the trajectory endpoints were positioned in multiple CT slices, the respective axial pCT slices were intensity-wise cumulated, and trajectory was overlaid on it, as shown in Figure 6—Column 5.

In addition to 2D directional angle errors, head and tail end displacements between the actual and the registered trajectories were measured by overlaying trajectory points on intraoperative radiographs. The marker length difference was measured between the estimated trajectory length and the marker’s ground truth length value. On global optimal registration, these metrics should result in a zero value, signifying that the entire vertebra is registered optimally without any misalignment between the AP and lateral planes. Registration errors together with the screw head end blocking a portion of the marker in the AP direction resulted in a nonzero trajectory length difference.

## 6. Discussion

Due to vertebral deformation between the intraoperative and preoperative environments, individual vertebra was subjected for deformable registration. Hence, though images possessing multiple vertebral levels were acquired once, different but closer registration parameters were estimated for individual vertebra. Under the postural spine deformations, such deformable registration practice are found to be locally rigid but globally deformable.

The framework bypasses the requirement of several image preprocessing techniques, such as filtering, image enhancement, feature extraction and segmentation processes, that are usually practiced in 3D-2D image registrations to minimize the intensity differences or for better feature mapping between the projected and real X-ray images. In addition, the ICP-DRR based anatomical landmark registration bypasses the requirement of computation intensive projection image generation during the optimization stage, hence supporting the framework’s applicability in the faster intraoperative applications assisting with clinically acceptable registration accuracy. Such landmark-based registration framework is suitable especially in surgical procedures wherein surgeons navigate surgical tools through referring to their intuitive 3D-2D landmark mapping knowledge.

The registration accuracy depends on corresponding 3D-2D anatomical landmark identification and the accuracy with which 3D-2D pose is estimated. The accurate landmark identification was challenging due to low-contrast intraoperative X-ray images and vertebral level missing EOV. Hence, during the landmark registration, it was imperative to minimize the error in mapping 3D-2D landmarks. When the vertebra level misses EOV, landmark registration improves after the right identification of corresponding control points subjected to different out-of-plane transformations. More often, vertebral centroids, pedicle centers and inferior ends of the spinous process were employed for landmark registration in the AP plane. Alternatively, when vertebral body endplates’ anterior and posterior edges were displayed, the corresponding landmarks control points were selected to register the landmarks subjected to out-of-plane rotations. A combination of many such landmarks with optimal 3D-2D mapping knowledge can be utilized to estimate individual vertebral poses in optimally matching projection images with the intraoperative radiographs acquired in various planes. Due to the defective shape of the L4 vertebral level, there were challenges associated with the fractured vertebra while registering in the lateral plane. The imprecise vertebral body corner points identification due to its fragmented body region resulted in moderate magnification factor registration.

Whenever vertebral endplates and pedicles miss the EOV projection in a real X-ray image, and when the head end of the screw blocks a portion of marker length in the AP direction; the trajectories in the AP and lateral directions majorly deviated from getting intersected. A study can be performed on these aspects by acquiring multiple oblique images.

## 7. Conclusions

The working principle of the designed framework naturally fits in line with the existing PSI surgeries that are currently practiced in local hospitals. The designed ICP-DRR based hybrid registration framework accurately mapped the pedicle screw trajectory between intraoperative X-ray images and preoperative CT images. The trajectory registration was completely dependent on anatomical landmark registration. On unclear visibility of anatomy, a combination set of multiple landmarks was identified for the optimal vertebral pose estimation. The estimation of marker length and 2D directional vectors can be further optimized by additionally referring to the oblique images. The generated DRR images after anatomical landmark registration clinically validated the estimated ICP registration parameters. The control point-based registration through bypassing the projection image generation process of intensity-based registration has made the registration process suitable for faster intraoperative applications. The patient specific registration framework such as ours is free from data requirement and generalizability issues associated with deep learning based 3D-2D registration frameworks. Automated landmark identification in both 3D and 2D planes along with parallel computation-based registration can further accelerate the registration process.

## Figures and Tables

**Table 1:** Distance between the unconnected trajectories at the head and tail ends are tabulated for the study dataset.

Vertebral Level	Head	Tail
T11	2.00 mm	1.00 mm
L1	0.00 mm	0.00 mm
L3	0.00 mm	1.00 mm
L4	0.00 mm	1.00 mm
L5	1.00 mm	5.00 mm

**Table 2:** Tabulation of trajectory registration error in the AP and lateral planes, and trajectory length error measurement between the actual and the registered trajectories.

Vertebral Level	AP—Plane			Lateral—Plane			Trajectory Length Error
	Head	Tail	Angle	Head	Tail	Angle	
T11	1.00 mm	0.00 mm	$6.91^{0}$	1.00 mm	2.00 mm	$1.54^{0}$	2.06 mm
L1	1.00 mm	0.00 mm	$2.64^{0}$	0.00 mm	1.00 mm	$0.45^{0}$	0.91 mm
L3	0.00 mm	1.00 mm	$3.67^{0}$	0.00 mm	0.00 mm	$0.00^{0}$	3.68 mm
L4	0.00 mm	0.00 mm	$0.00^{0}$	1.00 mm	2.00 mm	$5.18^{0}$	6.65 mm
L5	1.00 mm	3.00 mm	$11.50^{0}$	1.41 mm	3.16 mm	$2.75^{0}$	0.03 mm

## Data Availability

Not applicable.

## Appendix A. Dataset Specification

The CT and X-ray image clinical dataset specification is given in Table A1. The pCT images are acquired at peak X-ray generator voltage set in a range of 120 to 140 KVP and X-ray tube current set in a range of 200 mA to 300 mA. The C-arm X-ray generator voltage and X-ray tube current are in a range of 65 kV to 76 kV and 3.3 mA to 3.8 mA, respectively.

**Table A1:** Dataset specification includes resolution, image size and vertebral levels considered for the study purpose.

Dataset	CT Volume	CT Voxel Dimension	X-ray Image Size	X-ray Pixel Resolution
Patient 1 (T11 and L1)	512 × 512 × 243	0.56 × 0.56 × 3	1024 × 1280	0.23 × 0.23
Patient 2 (L3, L4 and L5)	512 × 512 × 242	0.47 × 0.47 × 3	576 × 576	0.35 × 0.35
